# Mechanical and Environmental Assessment of Lathe Waste as an Addiction to Concrete Compared to the Use of Commercial Fibres

**DOI:** 10.3390/ma16175740

**Published:** 2023-08-22

**Authors:** Jorge Los Santos-Ortega, Esteban Fraile-García, Javier Ferreiro-Cabello, Carlos González-González

**Affiliations:** Department of Mechanical Engineering, Mechanical Area of Continuous Media and Theory of Structures, University of La Rioja, San José of Calasanz, nº31, 26004 Logroño, Spain; esteban.fraile@unirioja.es (E.F.-G.); javier.ferreiro@unirioja.es (J.F.-C.); carlos.gonzalezg@alum.unirioja.es (C.G.-G.)

**Keywords:** fibres, concrete, waste, compression strength, flexural strength, life cycle assessment, sustainability

## Abstract

The use of fibres applied to concrete in order to improve its properties is widely known. Nowadays, research is not only focused on improving mechanical properties but also on the environmental implications. The aim of this research was a mechanical and environmental comparison between different types of fibres. For this purpose, commercial fibres of three materials were used: low carbon steel, modified polyolefins, and glass fibre. In order to improve the sustainability of the sector, we also analysed and compared the performance of using a waste product, such as fibres from machining operations on lathes. For the evaluation of the mechanical properties, compression and flexural tests were carried out. The results show that the use of low carbon steel fibres increases the flexural strength by 4.8%. At the environmental level, and in particular for impact categories such as the Global Warming Potential (GWP), lathe waste fibres prove to be the most suitable. For instance, compared to glass fibres, CO_2_ emissions are reduced by 14.39%. This is equivalent to a total of 38 kg CO_2_ emissions per m^3^ of reinforced concrete. In addition to avoiding the consumption of 482 MJ/m^3^ of fossil fuels, the results of the research indicate the feasibility of using waste fibres as a substitute for commercial fibres, contributing to an improved environmental balance without losing mechanical performance.

## 1. Introduction

Concrete is the primary material used in the construction sector. Nowadays, there is a burgeoning field of research on this material that endeavours to improve its mechanical and environmental performance. Nevertheless, concrete still presents some problems directly related to its behaviour and use. One of the most commonplace problems is hydraulic shrinkage cracking. This type of cracking occurs even at early ages and during the use phase. When a quantity of concrete is exposed to atmospheric conditions, its internal water content evaporates and thus the concrete’s dimensions shrink. During this process, internal stresses are generated by shrinkage. Consequently, if the internal shrinkage stress is higher than the internal strength of the concrete, the concrete cracks. Crack formation in reinforced concrete is a serious problem because it can lead to corrosion of the reinforcement [1]; on the other hand, cracks cause discontinuities in the material and a decrease in load resistance. One way of mitigating this problem is by adding fibres to the matrix during the raw materials mixing process. As the fibres are distributed homogeneously throughout the concrete, they behave like a structural micro-reinforcement, providing mechanical strength and ductility. This alleviates the aforementioned problem of early cracking.

In general, reinforced concretes usually use only one type of fibre; however, the simultaneous use of several types of fibres from different materials is called hybridisation, which produces synergies [2]. The current market for fibres for concrete is very broad and novel. However, the most common materials are plastics [3], steel fibres [4,5] glass fibres [6], and even natural fibres [7]. A particular fact is that the use of fibres at the microscopic level is being investigated more and more continuously, with promising results being obtained. In the specific case of plastic fibres, the research of Shi et al. stands out, where they evaluated the behaviour of cement mortar that includes carbon nano-fibres (CNF). The macroscopic mechanical strength results show an improvement of 28% in Young’s modulus and 34% in the flexural strength of the specimens [8]

Meanwhile, more and more authors are investigating how to use waste from various industrial processes as fibres. Some of these types of waste are polyethylene terephthalate (PET) from bottles [9], which would thereby improve mechanical and thermal properties. Some of the results obtained by Fraternali et al. show a decrease in the thermal conductivity coefficient of 18% and an increase in compressive strength of 35% [10]. Also, steel fibres from the recycling of worn tyres can be used [2,11,12]. According to Zhong and Zhang, when using recycled tyre steel fibres, there is a 116.8% increase in flexural strength, as well as a significant reduction in the problem of drying shrinkage of concrete [2]. These satisfactory results represent an alternative way to recycle tyres’ rims and reduce the impact of traditional fibre production [13].

Other wastes from the metal industry are shavings from material removal processes, which are produced by numerical control machines (CNC), lathes, milling machines, etc. It is estimated that, in one day, a conventional lathe produces a total of 3–4 kg of swarf. In addition, automatic machines such as CNC machines are estimated to produce 1200 Mt/year [14]. Therefore, the machining industry as a whole generates large quantities of waste, the majority of which are reused in smelting processes to generate new metals. However, this recycling process entails additional costs due to internal processes such as the pre-treatment of swarf through cleaning, given that swarf is covered with coolants such as oil and cutting fluid. Since chips are small in size and homogeneous in length, one possible use for this waste is to incorporate it into concrete. Malek et al. report increases in compressive strength of up to 36.3% when 15% of the fine aggregate is replaced with steel fibres from CNC machines [14]. Althoey and Akter also use lathe iron waste dust in proportions of 5% to 20% as a substitute for fine aggregate. They report increases in compressive strength from 4.75% to 38%, respectively, and for flexural strength an increase of 11% for an addition of 20% as fine aggregates replacement [15].

There are numerous past studies on using fibres in concrete and mortar. However, there is a clear lack of knowledge regarding the environmental impact of the use of fibres in concrete. There are only a few specific case studies where a structural element uses some type of fibre, such as slabs [16], beams [17], and reinforcement [18]. The research evaluated by Sbanieh et al. proposes the use of fibre-reinforced polymer as an external reinforcement in beams. The aim is to reduce the amount of steel from internal reinforcement. Among the results presented is that fibre-reinforced polymer tends to be more sustainable and environmentally friendly compared to traditional materials due to their durability and corrosion resistance [17]. In the same line of study is the research carried out by Inman et al., where they compare reinforcement bars made of basalt fibre-reinforced polymer (BFRP) against traditional steel bars in concrete beams. The results show that, for impact categories such as Global Warming Potential, there is a saving of 62% when using BFRP reinforcement instead of conventional steel bars [18]. As can be seen, previous research that has evaluated the environmental impact of the use of any type of fibre is in a particular case study. However, there is a lack of knowledge as to whether the use of steel, plastic, or glass fibres is better for the environment.

This gap in the research has motivated this study, which aims to examine the mechanical and environmental behaviour of using fibres in concrete. To this end, the most widely used fibres are analysed, such as low-carbon steel fibres, glass fibres, and polyolefin fibres, as well as a potential waste product—shavings from a lathe machining process. For the mechanical analysis, the behaviour of specimens subjected to compression and flexure tests is studied. In these tests, minimum, medium, and maximum fibre quantities are dosed. And for the environmental evaluation, a cradle-to-gate Life Cycle Analysis (LCA) is conducted. This facilitates drawing environmental conclusions regarding certain impact categories. This analysis provides new information on the impact of using one type of fibre or another in reinforced concrete. To better comprehend these impacts, the analysis is performed with 1 m^3^ of fibre-reinforced concrete and does not deal with structural elements or specific case studies. Therefore, it is possible to determine the impacts associated with using a specific type of fibre in a unit of volume, and thus be able to extrapolate the results to complex solutions such as structural elements and case studies. In this way, the sustainability of the sector can be improved and can be made more environmentally friendly

## 2. Methods Applied

This section describes the materials used to make the test specimens, as well as the procedures used to carry out the test (mechanical criterion), and the LCA to obtain the environmental results.

### 2.1. Raw Materials

This raw materials section describes the main characteristics of the cement, aggregates, water, and fibre materials used in the manufacture of test specimens.

#### 2.1.1. Cement

CEM II/A-L cement was used as a binder, as classified by regulation [19]. The amount of clinker in its chemical composition ranges between 80 and 94%, with 88% being the average. In addition, belonging to subcategory L, it has other components such as limestone (L) 12%. It has a medium–high strength of 42.5 MPa at 28 days. It is used in non-structural, reinforced, and mass precast concrete. Table 1 shows the rest of the physical, chemical, and mechanical properties of the cement used.

In order to know the amount of cement to use, the exposure class of the concrete must first be defined. This concrete has been modelled through the condition that it is in a medium humidity environment (called XC3). According to the Spanish regulations on concrete structures [20], a medium humidity environment (XC3) is one in which the concrete is in external contact with the atmosphere and subjected to the action of rainwater. For this type of exposure class, the minimum amount of cement to be used is 300 kg/m^3^, as well as water/cement (w/c) of 0.55.

#### 2.1.2. Aggregates

**Figure 1:**
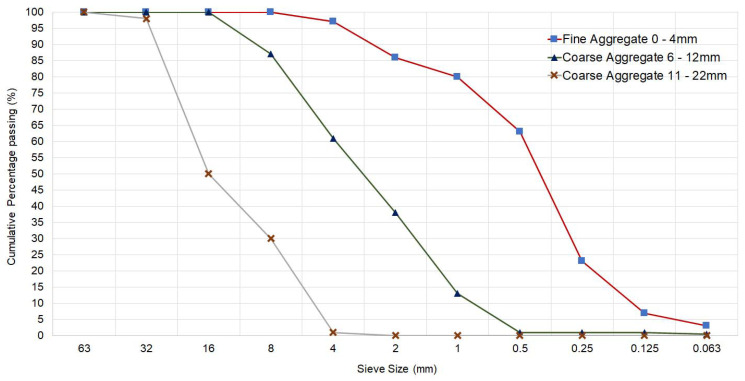
Fine aggregate and coarse aggregates granulometry.

The aggregates used are a mixture of fine and coarse aggregates. Washed sand with a size of 0–4 mm and a density of 2440 kg/m^3^ was used as fine aggregate. Washed gravel was used for the coarse aggregates. These coarse aggregates are composed of two types of sizes. The first coarse aggregate is gravel with a size of 6–12 mm and the second coarse aggregate has a size of 11–22 mm. Both coarse aggregates have a density of 2650 kg/m^3^. Figure 1 shows the cumulative percentage of fine aggregate and coarse aggregate through sieve analysis according to EN 933-1 [21].

The supplier provides a mixture of fine and coarse aggregates. The mix consists of 50% fine aggregates, 25% coarse aggregates of size 6–12 mm, and finally 25% coarse aggregates of size 11–22 mm. These percentages are by weight, which means that, for the production of 1 m^3^ of concrete, a total of 900 kg of fine aggregates, 450 kg of coarse aggregates of size 6–12 mm, and lastly, 450 kg of coarse aggregates of size 11–22 mm will be necessary.

#### 2.1.3. Water

The water used to make the test specimens is industrial water, with a pH of 8.2 and sulphates with a value of 0.272 g/L. Table 2 shows the other properties of the water. Given the w/c ratio and the minimum amount of cement indicated by the regulations depending on the type of exposure class [20]. The amount of water required in 1 m^3^ of concrete is 165 kg of water.

#### 2.1.4. Fibres

In this research, fibres from four materials are used: low carbon steel, modified polyolefins, glass fibres, and lathe waste fibres. The first three types of fibres are commonly used as additives in concrete and the lathe waste is being evaluated for its possible use. The following sections explain the characteristics of the different fibres used.

##### Low Carbon Steel Fibres

**Figure 2:**
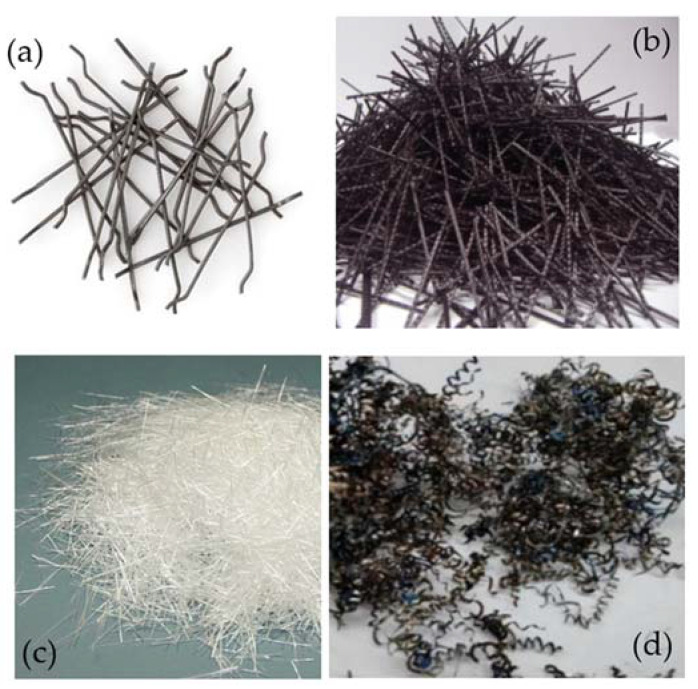
Fibres used in this study. (**a**) Low carbon steel fibres; (**b**) Modified polyolefins fibres; (**c**) 12 mm and 36 mm glass fibres; (**d**) Lathe waste fibres.

These are fibres formed from cold drawn low carbon steel wire. They have a high tensile strength (1200 MPA ± 15%) and ductility at break. Their use is indicated for concrete with strengths of 25 or 30 MPa. In addition, the ends of these fibres have an anchoring system that enhances the behaviour between the concrete and the fibre, guaranteeing much better anchorage than simple friction between fibre and matrix. Their field of application is structural reinforcement in concrete for paving and foundation slabs, and shotcrete. A sample of the steel fibres used is shown in Figure 2.

##### Modified Polyolefins Fibres

This fibre is composed of modified polyolefins (homopolymer polypropylene and polyethylene). Their advantage is that they are not affected by oxidation and corrosion processes. They have high tensile strength (400 MPa ± 5%). They are used in shotcrete in tunnels, mining, as well as in screeds, floor slabs, paving, and prefabricated elements. It has been observed that their durability against chemical attacks makes them ideal for offshore platforms and safety structures. An example of modified polyolefins fibres is shown in Figure 2.

##### Glass Fibres

These alkali-resistant glass fibres are utilized in concrete and mortars to reduce cracking and increase their impact resistance and durability, while also augmenting tensile and compressive strength. They are characterized by a high breaking strength (1650 MPa). Their applications are diverse, ranging from concrete pavements, prefabricated elements, sprayed concrete and mortar, slabs, floor slabs, etc. In this study, two types of glass fibres are used that differ in length. One measures 12 mm and the other 36 mm; meanwhile, their physical-chemical composition is the same. These lengths of glass fibres are the most commonly used as additives in concrete, so it was decided to study them. Figure 2 includes an image of the glass fibres used.

##### Lathe Waste Fibres

The so-called lathe waste fibres are the waste generated by the machining operations of parts by metal removal. As this is a non-automated process, the waste chips produced vary in dimension. The fibres used in this work have a variable cross-section, between 0.3~1.5 mm^2^, and their length ranges from 10~70 mm. The lathe waste fibres used come from the machining of S235 steel parts. For this purpose, it is assumed that, if the source material has better strength than the one used in this research (S235 steel), better results will be obtained in the flexural and compression tests. On the other hand, a material with deficient mechanical performance can lead to a reduction in the strength of the reinforced concrete. Figure 2 shows the lathe waste fibres collected in this study. This residue has great potential, as previous research has demonstrated that adding it to concrete leads to an increase in flexural strength of 7.1% for 5% additions and an increase in tensile strength of 4.2% for the same doping value [14]. Furthermore, this material exhibits a new potential use, instead of recycling it to obtain low carbon steels. Table 3 shows the mechanical and geometric properties of the fibres used.

#### 2.1.5. Fibres Dosages

A common dosage criterion was established for all fibres. Depending on the manufacturer and the type of fibre, a range of minimum and maximum addition values is determined that differs from fibre to fibre. Three dosage values were proposed, the first one being the minimum, followed by medium and maximum. To obtain the values according to the range, an economic criterion was established. The prices of electro-welded mesh (€/m^3^) of diameters Ø6, Ø8, Ø10 mm necessary to make 1 m^3^ of concrete were taken as a reference. This data were obtained from the price database of the structural calculation programme CYPE [27], as well as from the market prices of the various fibres (€/kg fibres). Based on these data, the quantities of kilograms of fibres required per m^3^ of reinforced concrete (kg fibres/m^3^) were established in terms of minimum (MIN), medium (MED), and maximum (MAX) addition. These values are listed in Table 4.

#### 2.1.6. Samples Assessed

Table 5 shows the composition of the mixes used to produce the test specimens. As can be seen, all the mixes have the same water–cement ratio (w/c) of 0.55, as well as the same amount of aggregates. Meanwhile, as mentioned above, several test specimens are analysed. The reference test specimens (S_REF) do not have any type of reinforcement fibre; S_MIN are the test specimens that incorporate the minimum amount of fibres. S_MEDIUM are those whose quantity of fibres is the median of the acceptable doping range. And finally, the S_MAX specimens are those configured by adding the maximum amount of fibres possible.

This section explains the different procedures applied in this study. Firstly, the mechanical criterion is explained, that is, how the test specimens doped with the various types of fibres were created, from the mixing process of the components to their testing on the machines. For the environmental analysis, on the other hand, the LCA is utilized, which quantifies the environmental impacts associated with a product or service.

### 2.2. Mechanical Criterion

The first step in performing mechanical tests on concrete is to create test specimens. In this study, two specimens of different dimensions were used—specimens of 600 × 150 × 150 mm for the flexural test and specimens of 150 × 150 × 150 mm for the compression test. The UNE-EN-83151-1 standard [28] was followed for this test, which advises stopping the concreting process in temperature conditions below 0 °C, since the cold can have a negative effect on the setting process.

For the correct mixing of the concrete, the fibres were added constantly and in small quantities. In this way, a uniform distribution was achieved in each mix. Subsequently, the concrete was poured into the moulds, taking into account the temperature at the time of pouring [20], and after the concrete was vibrated. The curing process varies between 24 and 72 h. Finally, the specimens were submerged in a water chamber at a constant temperature of 20 °C for 28 days. Once the curing time had ended, the respective mechanical characterization tests were carried out.

**Figure 3:**
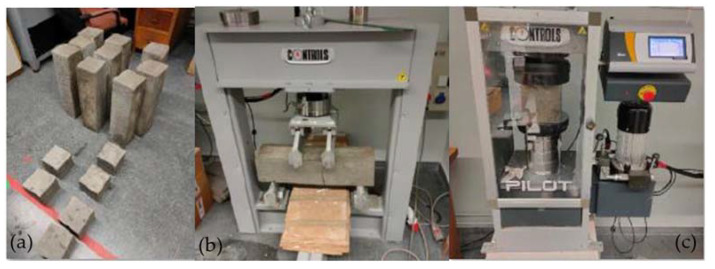
(**a**) Test specimens. (**b**) Flexural test. (**c**) Compression test.

The mechanical tests were carried out in accordance with the UNE-EN 12390-5 [29] standards for the 600 × 150 × 150 mm specimens, which were subjected to flexural tests. In this case, three tests were carried out on each type of fibre. The guidelines established in the standards indicate the loading speed. This must be between 0.04–0.06 MPa/s with a margin of ±1% in the speed of load application until breakage occurs. For the case of this study, a value of 0.05 MPa/s was selected. The UNE-EN12390-3 standard [30] establishes the criteria for compression tests. The specimens were 150 × 150 × 150 mm and a total of four tests were performed for each type of fibre. In the compression test, the range was 0.2–1 MPa/s and the rate of increase was ±10%. Figure 3 shows the specimens tested, as well as the machine used in the flexural and compression tests.

### 2.3. Life Cycle Analysis

The environmental assessment is completed through LCA, which is a tool for determining the environmental impacts associated with a product or service. It is regulated by the ISO 14040 [31] and ISO 14044 [32] standards, which establish a series of stages.

#### 2.3.1. Scope and Goals

The objective of the environmental study is to determine the impacts associated with incorporating different types of fibres into concrete. In this way, the environmental impacts of using fibres of different materials can be determined. First, the scope of the LCA study must be defined. It is known from previous literature that the greatest environmental impacts are produced in the concrete production phase [33] compared to later phases of assessment such as construction, use, maintenance, and demolition recycling. In these latter phases, it is assumed that fibre-reinforced concretes of different materials will perform identically, so that the impacts they generate will be the same in all studies, with no differences between them. Therefore, in this research, the scope of LCA is limited from cradle to gate.

#### 2.3.2. Functional Unit

The functional unit selected for this research is 1 m^3^ of fibre-reinforced concrete. This unit is selected because prior studies [34,35] have worked with this same unit. It is also a unit widely used in civil engineering and construction.

#### 2.3.3. System Boundary

**Figure 4:**
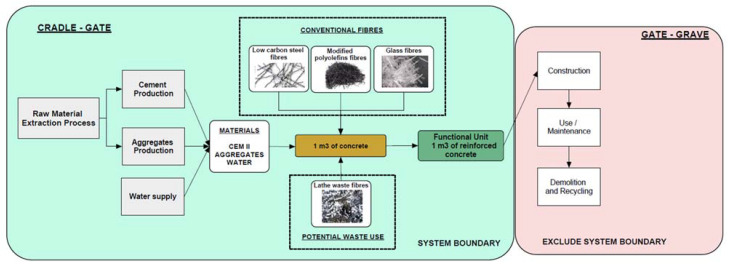
System boundary for fibre-reinforced concrete.

Figure 4 shows the system boundary of the LCA of this research. As can be seen, this LCA includes the production processes of the raw materials that make up concrete. These are the production of cement, aggregates, and water. The manufacturing processes of the three most commercial fibres have also been defined: low carbon steel fibres, modified polyolefin fibres, and glass fibres. For lathe waste fibres, as it is a waste and there is no specific process for its modelling, it was decided to create a specific process for it. Usually, this type of waste from the manufacture of pieces by chip removal is considered scrap and, for a posterior use of this waste, it must be recycled. The recycling process consists mainly of melting the scrap in an electric arc furnace. This furnace consumes electricity from the electricity grid. Therefore, by using this waste as fibres in concrete, the waste is prevented from being melted down in a furnace. Therefore, the process of lathe waste fibres is modelled as avoiding the melting of scrap in a conventional furnace.

#### 2.3.4. Life Cycle Inventory (LCI)

The LCI tries to determine the flows of matter, energy, waste, emissions, etc., involved in the process of creating the functional unit. These factors are key to developing an LCA because an inaccurate quantity of an element comprising the functional unit can lead to erroneous environmental results and their respective interpretation.

Nowadays, a wide range of software is available to perform LCAs. This study has opted for the SimaPro 9.4 software [36]. This programme contains a number of databases with predefined processes and elements. The database chosen was the ELCD (European Life Cycle Database) [37] as well as the Ecoinvent v.3 database [38].

The quantities of the raw materials used are displayed in Table 6. These quantities are those previously obtained from the dosage criteria used, for the production of 1 m^3^ of reinforced concrete.

#### 2.3.5. Life Cycle Impact Assessment

Once the quantities required to produce 1 m^3^ of concrete have been defined. At this stage, the impacts resulting from the use of elements defined in the LCI are assessed. Firstly, an environmental methodology must be chosen. A broad literature of environmental impact methodologies is now available. However, the choice of methodology is an important issue. It is necessary to choose the assessment methodology that best fits the case study and that contains impact categories that can be significantly affected by the chosen functional unit. For the specific case of building elements (mortars, concretes, precast elements etc.), the environmental assessment methodology EPD 2018 is used, because this methodology is used to make so-called Environmental Product Declarations (EPD). These EPDs make it possible to compare the environmental impacts between specific products or elements of the same type. For example, this methodology is used in previous studies to compare steel-reinforced concrete beams, reinforcement steel, and pouring concrete [18]. In this research, the comparison of 1 m^3^ of concrete reinforced with fibres of different materials is the one that best fits. In this way, it will be possible to compare which fibres produce fewer environmental impacts in the impact categories included in this methodology. These impact categories are shown in Table 7.

## 3. Results and Discussion

This section shows the results obtained from this research and their discussion. These are divided into mechanical results obtained directly from the flexural and compression tests and, on the other hand, the environmental results determined by the LCA.

### 3.1. Mechanical Results

#### 3.1.1. Compression Test Results

**Figure 5:**
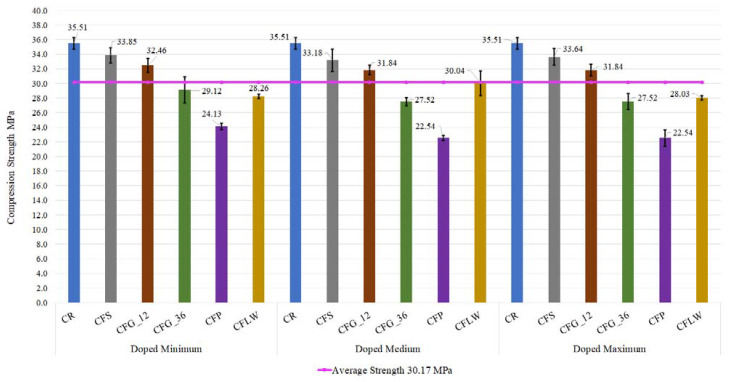
Results of compression test according to doping level and type of fibre.

The first results to be discussed are those of the compression test. Figure 5 shows the compressive strength values at 28 days, depending on the amount of fibre used (Minimum, Medium, Maximum) and the nature of the fibre, as well as the standard deviation bars for each specimen. Table 8 shows the numerical values of the standard deviation. The nomenclature for the alternatives according to the nature of the fibre is: concrete reference (CR), concrete fibres steel (CFS), concrete fibres glass 12 mm (CFG_12), concrete fibres glass 36 mm (CFG_36), concrete fibres polyolefins (CFP), and concrete fibres lathe waste (CFLW).

As can be noted, the reference specimens have an average value of 35.51 MPa and, on the other hand, the purple line shows the average strength of all the tests, reaching a value of 30.17 MPa. Let us begin by explaining the results for the steel fibre. As one can see, the results are practically the same regardless of the percentage of doping, and, in comparison with reference concrete, a 5.5% decrease in strength is obtained. In comparison with the average test results, however, steel fibre performed better in terms of strength, increasing by 9.97%. Steel fibres obtained the best results in terms of compressive strength. Compared to the literature, there is a discrepancy in the use of steel fibres. For example, Caggiano et al. observed a percentage increase in compressive strength of 15% compared to reference concrete when using steel fibres at a ratio of 60 kg/m^3^ [4]. This difference in results between the two studies is due to the number of dosages proposed. If analysed in the case of the current research, for the maximum level of doping, the use of a quantity of steel fibres of 14.84 kg/m^3^ was proposed, which, in comparison with the dosage of 60 kg/m^3^, represents a percentage reduction of 75.3%. Other authors, such as Martinelli et al., obtained similar results to those of the current research, where they did not observe an improvement in the compressive strength of concrete reinforced with steel fibres with the fraction volumes they used. The compressive strength is mainly controlled by the property matrix [39]. The standard deviation results show a higher deviation for medium dosages, where it reaches a value of 1.51 MPa. In contrast, for minimum dosages, the deviation is 1.08 MPa and for the maximum dosage it is 1.12 MPa. Therefore, the average standard deviation is 1.23 MPa. This value is 36.67% higher than the average standard deviation of all the specimens, which is 0.9 MPa. Despite this great variability that may exist in the results, it is demonstrated that the steel fibres have the best behaviour in terms of compressive strength.

In the results for glass fibres, a distinction must be made between fibres measuring 12 and 36 mm. In the case of 12 mm fibres, as before, higher dosage levels resulted in lower compressive strength. This is because excessive doping reduces the compressive performance of the specimen. It is important to note that the results are above the doped specimens’ average by 6.22% and below the results of the reference concrete by 9.8%. In the case of 36 mm glass fibres, the same trend exists, but with worse results. This can be explained by the fact that large fibre lengths do not favour compressive strength. Regardless of the level of doping, the compression results are below average for the 36 mm glass fibres.

Meanwhile, polyolefins fibre obtained the worst test results. The reduction in compressive strength capacity is significant at 35.1% that of the reference concrete and 23.5% compared to the average. However, its average standard deviation (regardless of the amount of polyolefins fibre in the specimen) is 0.64 MPa. This is the lowest standard deviation and a reduction of 28.29% with respect to the total average. This reaffirms that its bad behaviour and loss of strength is not due to a bad execution of the tests because the variability of the results is small. It is indicated that this type of polyolefin fibre is not suitable for use in concretes that are going to be subjected to high compressive stresses. Their use is reserved for giving the concrete greater durability and flexibility in cracking under low stresses. The reduction in compressive strength when using plastic fibres is well known. Previous studies already indicate that an increase in the volume fraction of polypropylene fibres in concrete leads to a decrease in compressive strength between 1.22% and 38.7%. Among the answers to this decrease in strength may be that the increase in fibres leads to agglomerations when the critical dosage is exceeded, as well as to an increase in porosity and a decrease in compactness in the internal structure of the concrete [2].

Finally, the last fibre to be analysed is the lathe waste fibre. The results obtained are very satisfactory given that the results for medium doping are within the average strength. Moreover, its results are better than, for example, those of polyolefins fibres and 36 mm glass fibres, with an increase of 33.27% over polyolefins fibres and 9.16% over 36 mm glass fibres. As for their deviation analysis, it is worth noting that the specimens with lathe waste fibre have the second lowest variability of all the fibres, with a mean standard deviation of 0.76 MPa. This lack of significant deviation in the variability of the results gives the lathe waste fibres an advantage in their future application. They do not show extreme behaviour between tests. Their low standard deviation is due to the fact that lathe waste fibres have a spiral shape, and therefore their adhesion and cohesion surface is larger. This means that they bind more precisely to the concrete matrix, compared to other fibres where their bonding surface is lower, e.g., glass fibres or steel fibres.

In the lathe waste fibres with respect to the reference concrete, the decrease of the characteristic strength is 15.40%. The research evaluated by Malek et al. states that several authors have obtained considerable improvements in compressive and flexural strength in concrete doped with lathe waste [14]. However, these studies use lathe waste as a substitute for aggregates [40,41] in various proportions and not as an additional material. Therefore, the amount of lathe waste in proportion to 1 m^3^ reinforced concrete is much higher, giving it higher strength. The explanation for the characteristic reduction in strength when lathe waste fibres or steel fibres are incorporated is due to a very important aspect of the use of fibres, that is, their orientation and homogeneity within the matrix once the concrete has been cured [42]. Poor orientation means that the fibres offer no load bearing benefits and may even incur negative effects; this ultimately diminishes mechanical performance. Since the fibres are metallic, a possible solution could be to reorient the fibres in the desired direction using an electromagnet.

As a general comment on the compression test, the best results tended to be obtained with the lowest dosages, with the results decreasing as the number of fibres in the concrete increased. This fact is corroborated by other authors [43]. This tendency was evident in all types of fibres, except for the lathe waste fibres where the best results were obtained for medium doping. This observation can be reaffirmed by analysing the average standard deviations per dosage (independent of the type of fibre). For example, for the case of a minimum dosage, its standard deviation is 4.18 MPa with respect to the average of 30.17 MPa. This deviation increases to a value of 4.60 MPa for medium dosages and finally for maximum dosages to a value of 4.74 MPa. This results in an increase of 11.81%. Therefore, it is affirmed that the higher the amount of fibre to be used in the specimen, the greater the variability of the results and, consequently, the worse the results obtained.

In conclusion, the compression test shows the following decreases in strength with respect to the characteristic strength of the reference concrete, for the case of minimum dosages. Steel fibres lead to a decrease in strength of 4.46%. Glass fibres with a length of 12 mm reduce the compressive strength by 8.57%, as do glass fibres with a length of 36 mm with a percentage reduction of 17.98%. As for polyolefin fibres, this reduction value is 32.04% and for the lathe waste it is 20.40%.

#### 3.1.2. Flexural Test Results

**Figure 6:**
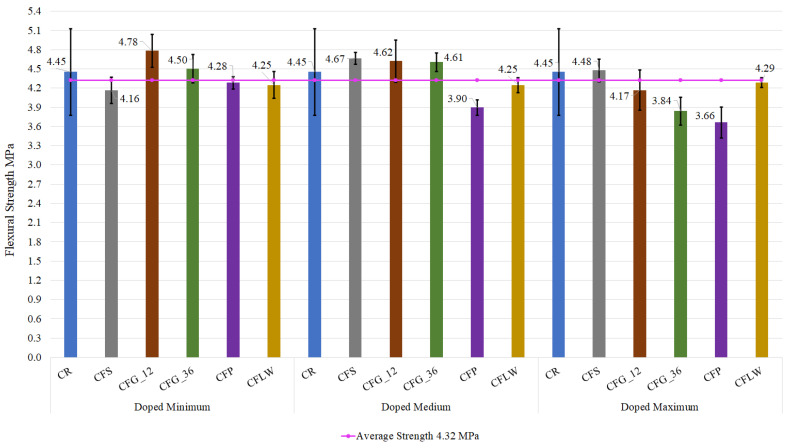
Flexural test results according to doping range and type of fibre.

Now that the results of the compression test have been described, the results of the flexural test are analysed. The values obtained are shown in Figure 6, and Table 9 presents the standard deviations for the flexural test.

In general, the flexural test results at the age of 28 days do not differ significantly from those of the reference concrete of 4.45 MPa; all test results tend to fall along the midline of 4.32 MPa. It is evident that the standard deviation is quite small for all samples. For example, the mean for the minimum dosages is 0.22 MPa, for the medium dosages 0.29 MPa, and for the maximum dosages 0.33 MPa. This results in an average standard deviation of 0.28 MPa. This reduced variability of the values of the results shows that, in the flexural test, the behaviour of the fibres is better than in the compression test. This is because the fibres in the concrete matrix provide greater ductility. In addition, their location perpendicular to the flexural stress results in a better performance compared to a reference concrete. This is due to geometry of the flexural test specimens. They have a larger lateral surface area than the opposite faces. This makes it more likely that the fibres will be oriented perpendicular to the flexural stress, providing strength.

As in the previous section, the same order is followed to describe the results of the different fibres, addressing steel fibres first. For medium and maximum dosages, the flexural strength exceeded that of the reference concrete. The maximum increase occurred at medium doping with an increase of 4.8%. This is why the flexural test illustrates the improved performance of doped concrete in tensile and flexural strength. The steel fibres have an anchoring system at their ends. This achieves a joint behaviour of the concrete matrix and the fibre. Plasticization of the hooks is necessary for tensile stressing to occur. This achieves a much higher anchorage than can be achieved by simple friction between the fibre and the concrete matrix. In addition, steel fibres with hooks increase the toughness of the matrix once the cracking process has started, contrary to what happens for a reference concrete [39]. The results obtained for a minimum dosage may be due to heterogeneity of the mix and poor distribution of the steel fibre. This leads to an average reduction of the flexural strength by 6.51%. This is equivalent to a reduction in strength of 0.29 MPa, which is an acceptable value.

As for the 12 mm glass fibres, for minimum and medium doping, the flexural strength values surpassed those of the reference concrete. The best result is at minimum doping with an increase of 6.9%. This improvement can be explained by the fact that this type of polypropylene fibre fosters more elastic behaviour in concrete, which is favourable for bending stresses. The same results are extrapolated for glass fibres, although the increase in flexural strength is now 2%. As explained above, for maximum dosage, the results decrease. This is due to the incorrect dispersion of the fibres in the specimen, giving rise to excessive concentrations in some points of the matrix and highlighting that this type of fibre, like polyolefins fibres, does not have any fixing element to the concrete matrix; their fixing system is only by simple friction.

The worst values were obtained for polyolefins fibres, with all doping levels below the average of the test results and the reference concrete. The minimum doping level gives the best results with a decrease in strength of 1% in comparison to the testing average and 3.8% of that of the reference concrete. As with the compression test, high dosages lead to excessive porosity and a reduction in the integrity of the matrix in the case of plastic fibres. This is evident in the research of Zhong et al., where for high dosage values they obtained a loss in flexural strength of 21.7% [2]. It should be noted, however, that although the decrease in strength is visible, this decrease is very small compared to the average of 4.32 MPa. In addition, the results of each specimen show a negligible standard deviation, as in the case of a minimum dosage where the deviation is only 0.10 MPa. It is clarified that the use of polyolefin fibres is only useful for reducing the effects of hydraulic shrinkage of the concrete at early ages. Consequently, when the concrete is subjected to compressive and flexural stresses, no improvement is reflected.

Finally, lathe waste fibres exhibit moderate behaviour. The three dosages behave very similarly and so the same result was obtained. Therefore, the results were slightly different from the average with a value 1.4% lower and 4.3% lower as compared to the reference concrete. The observation that can be made is that the doping level for this waste is irrelevant as it behaves similarly regardless of the doping level. For example, the waste fibres have a standard deviation of 0.10 MPa for minimum dosages, a value of 0.12 MPa for medium dosages, and 0.08 MPa for maximum dosages. Of all the fibres evaluated, those from the lathe waste have the lowest variability in their results, as can be seen in Figure 6. This is an advantage, since, for example, to achieve the same results in terms of strength, the lathe waste requires a smaller quantity of material.

In summary, the flexural test shows the following increases or decreases with respect to the flexural strength of the reference concrete, for the case of medium dosages. The steel fibres increase the strength by 4.79%. Glass fibres with a length of 12 mm increase the compressive strength by 3.81%, as do glass fibres with a length of 36 mm with a percentage increase of 3.44%. As for the polyolefin fibres, they indicate a percentage reduction of 12.50% and, finally, the lathe waste leads to a reduction in flexural strength of 4.64%.

In general, it should be noted that the behaviour is similar to that of the compression test, in which an increase in the number of fibres decreased the compressive strength. A greater number of fibres implies that, in the matrix of the specimen, there is an unevenness in the distribution of the fibres and therefore areas where the fibres are considerably concentrated. In this case, with minimum doping values, better results are obtained with the exception of the lathe waste, where the behaviour is practically homogeneous. This is due to the fact that the fibres are oriented in the direction of the stress [42], and therefore there is not so much dispersion in the results. In addition, as it is a material that has not been manufactured under fixed dimensions, this implies that fibres of different lengths and that are not excessively long are produced. This facilitates its uniform dispersion within the concrete matrix. Additionally, it is a type of fibre that provides higher levels of adhesion due to its geometry. Derived from its geometry, it is an anchoring system that improves the adhesion behaviour between the fibre and the matrix. This anchoring system is similar to that found in steel fibres. Therefore, it can be concluded that those fibres which make some kind of improvement for their fixation in the concrete matrix will obtain better results.

If the fibres are compared with each other, it is found that none of them are as effective in the compression test as they prove to be in the bending test. Among them, the best results are provided by steel fibres for both compression and bending, although 12 mm glass fibres behave similarly. As for waste fibres, their mechanical viability is evident when they are incorporated into fibre-reinforced concrete. Although there is a decrease in strength (compression and flexural) as compared to the reference concrete, it is not very significant, and in addition the values are practically within the average values obtained from the tests.

The lathe waste fibres show the least variation in terms of their doping level. As for the 36 mm glass fibres, their behaviour is similar to that of the 12 mm glass fibres but with worse results. And finally, the polyolefins fibres had disastrous results, which considerably limits their field of application, and the decreases displayed in their performance compared to the reference concrete must be afforded due consideration.

### 3.2. Environmental Results

**Figure 7:**
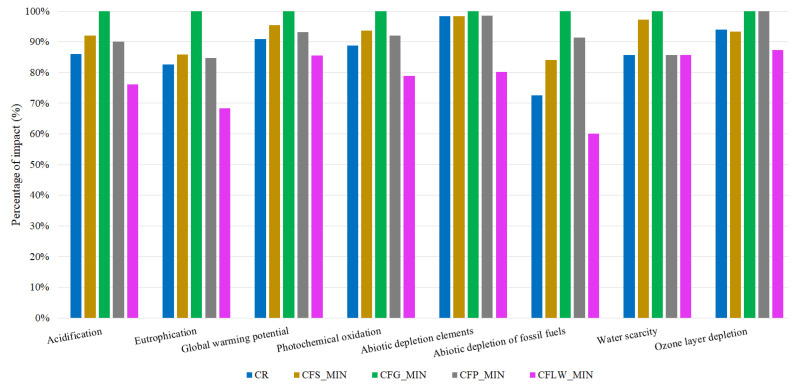
LCA results for 1 m^3^ fibre-reinforced concrete alternatives.

This section analyses the results obtained from the LCA. The impacts according to type of fibre are shown in Figure 7. For the 12 mm and 36 mm glass fibres, the results are combined into one option since the quantities to be doped in the fibre-reinforced concrete are the same. In addition, only the minimum dosages are analysed since the mechanical analysis proved that these offer the best results. And furthermore, the environmental impacts of medium and maximum dosages would result in greater quantities of material and, consequently, higher impact values.

With the exception of the reinforced concrete with lathe waste fibres, the rest of the fibre-reinforced concretes have greater environmental impacts than the reference concrete. This is due to the fact that the fibres, regardless of their nature, involve a prior manufacturing process. These processes consume raw materials, fuels, electricity, etc., thus a series of environmental impacts are generated. Among the various types of fibres, glass fibres have the greatest impact. This is because the material is derived directly from crude petroleum. It undergoes a series of intermediate processes to achieve polymerisation. It is estimated that the thermal energy required to produce 1 kg of polypropylene is 1.8 MJ [44]. In addition, this thermal energy comes directly from the electricity grid, which includes the impacts of transport losses and production emissions (from both renewable and non-renewable energy sources). This fact is reflected in the abiotic depletion elements category, where there is a 1.52% increase in impact compared to steel fibres and a 1.44% increase compared to polyolefins fibres. It is important to note that glass and steel fibres are also important sources of impact generation. As noted above, steel fibres involve the consumption of the raw materials that make up steel, such as iron. Iron is obtained from mining processes wherein water consumption is high. This fact is reflected in the water scarcity category wherein steel fibres have the second highest impact with a value of 97.28%. In addition, mining also affects soil acidification processes with an impact value of 92.13% and abiotic depletion elements with 85.93%. Therefore, steel fibres are ranked second in terms of environmental impacts. And finally, polyolefins fibres as well as glass fibres are derived from crude petroleum polymerisation processes. Due to origins of their raw material, polyolefins and glass fibres together constitute the solution with the third largest impact, coming in first in categories such as eutrophication and abiotic depletion elements and even equalling glass fibres in impacts in the ozone layer depletion category, because the process of refining crude oil emits greenhouses gases.

The next interpretation is to determine how much the environmental impacts increase with respect to the reference cubic metre of concrete. In this case, an average increase in environmental impact of 5.17% is observed for steel fibres, 12.60% for glass fibres, 4.58% for polyolefin fibres, and in the opposite case, an average reduction of −9.57% for lathe waste fibres. It can be seen that glass fibres have the greatest environmental impact when used in concrete. These results are in line with the current literature. The research by Gillani et al. evaluates environmentally the possibility of incorporating nylon waste fibres into concrete together with demolition aggregates. The authors indicate that nylon fibre waste produces lower environmental impacts (0.066 kg CO_2_) than the use of steel fibres (2.65 kg CO_2_), glass fibres (2.04 kg CO_2_), or polypropylene fibres (1.85 kg CO_2_/kg). This is justified by the fact that the treatment process of nylon fibres does not require processing of raw materials, but an efficient recycling process [45]. Furthermore, another remarkable comment between the two investigations is that, in the case of the research by Gillani et al., the environmental impacts of nylon waste fibres are practically zero with an impact of less than <1% even when larger quantities of fibres are used. This is in contrast to the impact values obtained in this research, where in the case of glass fibres they account for 12.60%. This difference in percentages is due to the fact that the production processes of virgin fibres involve processes of the transformation of raw materials and therefore greater consumption of resources as opposed to a recycling process.

In conclusion, the use of steel, glass, and polyolefin fibres has a major environmental impact on reinforced concrete. The current trend is to use waste as a substitute for these fibres. In this scenario, using recycled plastics as reinforcement fibres in concrete applications produces environmental and economic benefits [46]. For example, Yin et al. compared four environmental scenarios for the reinforcement of a footpath. One of the scenarios is the use of polypropylene fibres from an industrial recycling process. The results show that the use of industrially recycled polypropylene fibres results in a reduction of impacts compared to the other reinforcement alternatives. Compared to the usual alternative of steel reinforcement mesh, the production of recycled industrial polypropylene fibres saves 93% of CO_2_ emissions, 91% of water consumption, and 91% of oil raw material [47].

The lathe waste fibres diminish environmental impacts even below those incurred by the reference concrete. This is because the fibres have been modelled in the software to avoid steel production from scrap waste in an electric arc furnace. Although recycling scrap through smelting avoids the process of extracting raw materials to produce steel, this does not imply that there is no energy consumption. Nevertheless, energy consumption is high in the scrap smelting process, although it is lower than the consumption registered in traditional oxygen furnaces to generate raw steel [48]. Therefore, the use of this waste as fibres in reinforced concrete prevents it from being reused in the generation of recycled steel and thereby avoids the consumption of materials such as minerals (iron, silica, carbon) and energy, etc. For instance, if you compare 1 m^3^ of reinforced concrete with glass fibres against 1 m^3^ of reinforced concrete with lathe waste fibres in the abiotic depletion of fossil fuels category, lathe waste fibres offer a reduction of 39.83%, which is equivalent to savings of 462 MJ/m^3^. Likewise, a reduction of 14.39% is achieved, which translates into savings of 38 kg of CO_2_ for each cubic metre of fibre-reinforced concrete manufactured. Furthermore, categories such as acidification, eutrophication, and ozone layer depletion are reduced by 23.80%, 31.67%, and 12.60%, respectively, as the emission of nitrogen oxides (NO_x_), carbon oxides (CO_x_), harmful particles, and sulphides are prevented. These gases affect the formation of acids that are subsequently deposited in the soil, modifying its acidity and affecting flora and fauna, as in the case of acidification. These compounds also react with polar stratospheric clouds, emitting active chlorides and bromides which, under the catalytic action of UV rays, cause ozone depletion [49].

**Figure 8:**
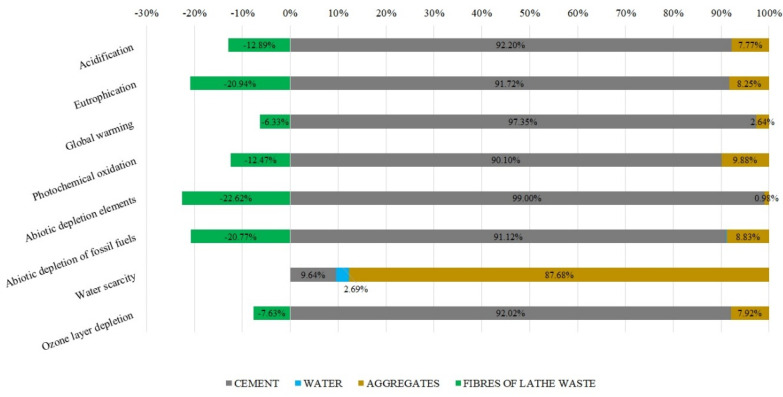
Environmental results of the lathe waste fibres alternative.

Figure 8 shows the specific results for the option incorporating lathe waste fibres. The different colours in Figure 8 indicate the values of each concrete component for each impact category. The lathe waste fibres have a positive impact, except for in the water scarcity category, where their impact is negligible (−0.12%). A negative result implies that using this product decreases the environmental impact. As mentioned above, the categories abiotic depletion elements, abiotic depletion of fossil fuels, and eutrophication had the best results with decreases of −22.62% (−1.62 × 10^−4^ kg SB eq/m^3^), −20.77% (−145 MJ/m^3^), and −20.94% (−0.010 kg PO_4_ eq/m^3^), respectively. These positive impacts are less significant in categories such as acidification −12.89% (−0.059 kg SO_2_ eq/m^3^), photochemical oxidation −12.47% (−0.05 kg NMVOC/m^3^), ozone layer depletion −7.63% (−5.19 × 10^−7^ kg CFC-11 eq/m^3^), and global warming potential −6.33% (−14.30 kg CO_2_/m^3^).

On the other hand, if the composition of 1 m^3^ of fibre-reinforced concrete is analysed, it is found that the most significant contributor to environmental impact is cement. This is because CEM II has a high percentage of clinker. To obtain clinker, large amounts of energy resources (transport, thermal energy in furnaces) and raw materials (limestone) are used. As a result, the cement raw material is highly affected. This is visible in categories such as abiotic depletion elements with a value of 99.0% (8.7 × 10^−4^ kg Sb eq) or the category of abiotic depletion of fossil fuels with an effect of 91.12% (768 MJ). Practically, the cement raw material is the main cause of environmental impacts. Other impact categories are also strongly affected such as acidification 92.2% (0.475 kg SO_2_ eq), eutrophication 91.72% (6.353 × 10^−2^ kg PO_4_ eq), global warming potential 97.35% (233 kg CO_2_ eq), photochemical oxidation 90.10% (0.408 kg NMVOC), and ozone layer depletion 92.02% (6.73 × 10^−6^ kg CFC-11 eq). Only in the water scarcity category is the impact of CEM II of 9.64% (12 m^3^) lower than that of the rest of the raw materials, with the exception of the water raw material. Its only appreciable impact value is in water scarcity with 2.69% (3 m^3^).

As for aggregates, as we have seen in previous sections, they affect ecosystem-related categories with greater intensity. The mining process involves large earth movements and high water consumption for the aggregate washing process. Therefore, land- and ecosystem-related categories are affected. For example, acidification 7.77% (4 × 10^−2^ kg SO_2_ eq), eutrophication 8.25% (5.71 × 10^−3^ kg PO_4_ eq), water scarcity 87.68% (111 m^3^), and abiotic depletion elements 0.98% (8.62 × 10^−6^ kg Sb eq). In order to carry out the aggregate extraction and processing processes, heavy machinery, such as large tonnage trucks and aggregate processing plants, must be used. Generally, this type of machinery is powered by fossil fuels. Therefore, the abiotic depletion of the fossil fuels category impacts with a value of 8.83% (74 MJ). The combustion of fossil fuels generates greenhouse gas emissions (COx, NOx) and particulate matter deposition. This process affects categories such as global warming potential 2.64% (6 kg CO_2_ eq), photochemical oxidation 9.88% (4.47 × 10^−2^ kg NMVOC), and ozone layer depletion 7.92% (5.79 × 10^−7^ kg CFC-11 eq).

Therefore, in this research, the environmental benefits of using lathe waste as fibres in concretes have been justified. However, the environmental benefits come from the consideration that this waste is not recycled by a scrap function process. Consequently, there are no environmental impacts resulting from it. This shows a possible knowledge gap, since, for example, it could also have been assumed that the use of these fibres can prevent this waste from being deposited in a landfill without any treatment and the possible associated impacts could have been studied. However, for this research it was decided to use the most common process for recycling machining waste, i.e., recycling through function.

## 4. Conclusions

In this research, three materials for fibres in reinforced concrete have been evaluated—steel fibres, glass fibres, and polyolefin fibres. In addition, the use of lathe waste as a fibre has been successfully evaluated. Its mechanical properties (compression and flexural) have been characterized and extensively evaluated in the current literature. However, the definition of their environmental impacts depending on the material of each fibre is highlighted. This results in the generation of new knowledge applicable to fibre-reinforced concrete. The main conclusions of this research are shown below.

In the compression test, the fibres evaluated did not show any improvement compared to the reference concrete. The best result is obtained by the steel fibres with a decrease in compressive strength of 5.5% with respect to the reference concrete (35.51 MPa). On the contrary, polyolefin fibres obtain a significant decrease in compressive strength with a decrease of 36.53%.

In the flexural test, a better performance response is evident compared to the reference concrete (4.45 MPa). These are an increase of 4.94% for the steel fibres in a medium dosage configuration and, on the other hand, a 7.41% increase in strength for 12 mm glass fibres with minimum doping.

In the case of lathe waste fibres, a reduction of 18.95% is obtained with respect to the compressive strength of the reference concrete and, in the flexural test, a reduction of 4.24%. However, the results obtained with this type of fibre are better than those obtained with polyolefin fibres.

In terms of dosage values, there is a clear tendency that the higher the amount of fibre added, the worse the strength results are. This is shown for all fibre types with the exception of lathe waste fibres, where the results show very little variability depending on the amount of doping, whether minimum, medium, or maximum. This is an advantage in favour of lathe waste fibres, there being a recycling option for this residue.

In the environmental results, and compared to unreinforced concrete, the environmental impacts increase by 5.17% for steel fibres, 12.60% for glass fibres, and 4.58% for polyolefin fibres. Lathe waste is the most environmentally viable option with a reduction in impacts of −9.57%. The maximum impact reduction is in the abiotic depletion elements category with −18.16%. For the global warming potential category, a reduction of −5.30% is found, which means that 14 kg of CO_2_ emissions are avoided for every cubic metre of fibre-reinforced concrete from lathe waste.

The use of polymeric fibres (glass fibres and polyolefin fibres) has a greater environmental impact. This is due to the fact that, during their manufacturing process, large amounts of raw materials as well as energy must be used in their internal processes. For instance, it is in the impact category of abiotic depletion of fossil fuels where the biggest differences exist. Glass fibres (irrespective of their length) occupy the first position, with an energy cost of 1160 MJ to produce 1 m^3^ of fibre-reinforced concrete. In second position are concretes reinforced with polyolefin fibres with a reduction of 8.62%.

When analysing the production of 1 m^3^ of concrete reinforced with lathe waste fibres, the best results are achieved, which are even below the values of the impacts produced by the reference concrete. This is visible in all evaluated impact categories where the use of lathe waste fibres results in positive impacts.

Among the values with the greatest positive impact are: −20.94%, −22.62%, and −12.89% in the categories of eutrophication, abiotic depletion elements and acidification, respectively, and−20.77% in the abiotic depletion of fossil fuels category, avoiding the consumption of a total of 145 MJ/m^3^.

The possibility of incorporating lathe waste as a fibre in reinforced concretes is successfully evaluated. Its environmental advantages compared to other fibres are the main support for its use.

## Figures and Tables

**Table 1:** Chemical composition of cement.

CEM II/A-L 42.5R			
		Regulation	Standard
Components	Clinker (%)	80–94	88
	Limestone (L) (%)	6–20	12
	Pozzolana (P) (%)	-	-
	Fly ash (V) (%)	-	-
	Steel slag (S) (%)	-	-
	Setting regulator, gypsum (%)	-	4
Chemical	Chemical sulfur trioxide (SO_3_) (%)	4 max	3.1
	Chlorides (Cl^™^) (%)	0.10 max	0.01
	Loss on ignition (%)	-	-
	Insoluble residue (%)	-	-
Physical	Blaine specific surface (cm^2^/g)	-	4.300
	Le Chatelier expansion (mm)	10 max	0
	Setting start time (min)	60 min	135
	Final setting time (min)	-	190
Mechanical	Compression day 1 (MPa)	-	20
	Compression day 2 (MPa)	-	32
	Compression day 7 (MPa)	-	-
	Compression day 28 (MPa)	42.5–62.5	53

**Table 2:** Physical and chemical properties of water.

Variable	Standard	Limits	Results
pH	UNE 83952 [22]	≥5	8.2 (g/L)
Sulphates	UNE 83956 [23]	≤1	0.272 (g/L)
Chloride Ion	UNE 83958 [24]	≤1 Prestressed	0.064 (g/L)
		≤2 Reinforced	
Carbohydrates	UNE 83959 [25]	0	0 (g/L)
Oil and grease	UNE 83960 [26]	≤15	0 (g/L)

**Table 3:** Comparative table mechanical and physical properties of fibres.

Specific Properties	Steel Fibres	Modified Polyolefins Fibres	Glass Fibres 12 mm	Glass Fibres 36 mm	Lathe Waste Fibres
Diameter	0.75 mm ± 10%	1.00 mm	0.020 mm	0.020 mm	-
Section	0.441 mm^2^	0.023 mm^2^	3.14 × 10^−4^ mm^2^	3.14 × 10^−4^ mm^2^	0.3~1.5 mm^2^
Length	35 mm ± 10%	46 mm ± 5%	12 mm	36 mm	10~70 mm
Tensile strength	1200 MPa ± 15%	400 MPa ± 7.5%	1650 MPa	1650 MPa	360 MPa
Density	7 g/cm^3^	0.91 g/cm^3^	2.679 g/cm^3^	2.679 g/cm^3^	7.85 g/cm^3^

**Table 4:** Fibres dosages per m^3^ of fibre reinforced concrete.

Amount of Fibres per m^3^ Concrete (kg/m^3^)					
Dosage	Steel	Glass 12 mm	Glass 36 mm	Polyolefins	Lathe Waste
Minimum	5.39	2.83	2.83	2.78	15.00
Medium	9.31	4.90	4.90	4.81	30.00
Maximum	14.84	7.80	7.80	7.66	45.00

**Table 5:** Composition of mixes (0.03 m^3^).

Sample	w/c	Water (kg)	Cement (kg)	Fine Aggregate (kg)	Coarse Aggregate 6/12 mm (kg)	Coarse Aggregate 11/22 mm (kg)	Steel (kg)	Glass_12 (kg)	Glass_36 (kg)	Polyolefins (kg)	Lathe Waste (kg)
S_REF	0.55	4.95	9.00	27.00	13.5	13.5	-	-	-	-	-
S_MIN							0.162	0.085	0.085	0.083	0.450
S_MED							0.279	0.147	0.147	0.144	0.900
S_MAX							0.445	0.234	0.234	0.230	1.50

**Table 6:** Materials dosages for 1 m^3^ of fibre-reinforced concrete.

Sample	w/c	Water (kg)	Cement (kg)	Fine Aggregate (kg)	Coarse Aggregate 6/12 mm (kg)	Coarse Aggregate 11/22 mm (kg)	Steel (kg)	Glass_12 (kg)	Glass_36 (kg)	Polyolefin (kg)	Lathe Waste (kg)
S_REF	0.55	165	300	900	450	450	-	-	-	-	-
S_MIN							5.39	2.83	2.83	2.78	15
S_MED							9.31	4.90	4.90	4.81	30
S_MAX							14.84	7.80	7.80	7.66	45

**Table 7:** Environmental impact categories covered by the EPD 2018 methodology.

Impact Categories	Unit
Acidification	kg SO_2_ eq
Eutrophication	kg PO_4_ eq
Global Warming Potential (GWP)	kg CO_2_ eq
Photochemical oxidation	kg NMVOC
Abiotic depletion elements	kg Sb eq
Abiotic depletion, fossil fuels (ADFF)	MJ
Water scarcity	m^3^ eq
Ozone layer depletion	kg CFC-11eq

**Table 8:** Standard deviation values for the compression test.

	Dosage	CR	CFS	CFG_12	CFG_36	CFP	CFLW
Standard deviation σ (MPa)	Minimum	0.81	1.08	0.96	1.79	0.44	0.31
	Medium	0.81	1.51	0.67	0.58	0.35	1.67
	Maximum	0.81	1.12	0.79	1.07	1.12	0.31
	Average	0.81	1.23	0.81	1.45	0.64	0.76

**Table 9:** Deviation standard values for the compression flexural test.

	Dosage	CR	CFS	CFG_12	CFG_36	CFP	CFLW
Standard deviation σ (MPa)	Minimum	0.67	0.21	0.26	0.22	0.10	0.21
	Medium	0.67	0.09	0.33	0.15	0.12	0.12
	Maximum	0.67	0.18	0.31	0.22	0.24	0.08
	Average	0.67	0.16	0.30	0.19	0.46	0.41

## Data Availability

Not applicable
